# How Can Viral Dynamics Models Inform Endpoint Measures in Clinical Trials of Therapies for Acute Viral Infections?

**DOI:** 10.1371/journal.pone.0158237

**Published:** 2016-07-01

**Authors:** Carolin Vegvari, Christoforos Hadjichrysanthou, Emilie Cauët, Emma Lawrence, Anne Cori, Frank de Wolf, Roy M. Anderson

**Affiliations:** 1 Department of Infectious Disease Epidemiology, School of Public Health, Imperial College London, London, United Kingdom; 2 Janssen Prevention Center, Leiden, The Netherlands; Centre of Influenza Research, The University of Hong Kong, HONG KONG

## Abstract

Acute viral infections pose many practical challenges for the accurate assessment of the impact of novel therapies on viral growth and decay. Using the example of influenza A, we illustrate how the measurement of infection-related quantities that determine the dynamics of viral load within the human host, can inform investigators on the course and severity of infection and the efficacy of a novel treatment. We estimated the values of key infection-related quantities that determine the course of natural infection from viral load data, using Markov Chain Monte Carlo methods. The data were placebo group viral load measurements collected during volunteer challenge studies, conducted by Roche, as part of the oseltamivir trials. We calculated the values of the quantities for each patient and the correlations between the quantities, symptom severity and body temperature. The greatest variation among individuals occurred in the viral load peak and area under the viral load curve. Total symptom severity correlated positively with the basic reproductive number. The most sensitive endpoint for therapeutic trials with the goal to cure patients is the duration of infection. We suggest laboratory experiments to obtain more precise estimates of virological quantities that can supplement clinical endpoint measurements.

## Introduction

According to a 2014 report, the costs of developing a new pharmaceutical has increased by 145% since 2003[1]. A major part of this cost increase is due to losses incurred on drug candidates that fail during clinical development[2]. Failure may be due to adverse events or an inability to detect beneficial effects on the target disease. The reasons why clinical trials fail to demonstrate efficacy of potential novel treatments are many and complex. Failure is often due to the choice of inadequate endpoints, or poor trial design. Good endpoints in clinical trials should fulfill the following two key criteria: i) they should reflect the action of the treatment on the underlying cause of disease, and ii) they should be reliably quantifiable.

Acute viral infections pose particular challenges for any assessment of the impact of novel therapies. They typically have a short incubation period (a few days) and rapid onset of clinical symptoms. Consequently, patients usually present at clinics after the viral load peak has passed and the infection is already in the decline phase. Only a few measurements of viral load or symptoms can be taken before the infection terminates due to the immunological response of the human host. Using the example of influenza A, we discuss how a quantitative analysis of viral population growth within the patient can help investigators to define more reliable endpoints and improve the assessment of the efficacy of potential novel treatments.

Symptom scores, although typically required by regulatory agencies as clinical endpoints, are seldom reliably quantifiable and often do not reflect the action of treatment on the pathogenic agent. For example, symptoms may be measured by asking patients how they are feeling, and therefore are at risk of being subjective and at best semi-quantitative[3]. Moreover, symptoms may not accurately reflect the pathologic process targeted by a specific treatment. In the case of influenza A, respiratory and systemic symptoms are not only caused by viral tissue damage, but also by the immune response to the viral infection itself. In severely ill patients with multiple morbidity-inducing conditions, symptom scores may not give any indication of the successful action of a treatment. Influenza treatment may clear the viral infection, but symptoms may persist due to secondary bacterial pneumonia[4].

The most suitable endpoints that best reflect the effect of a treatment on acute viral infections should be related to viral load. We chose influenza A as an example, because the recent controversy about the efficacy of established antiviral treatments against influenza A has reignited interest in improved endpoint measurements for the assessment of antiviral treatments and suitable statistical techniques to compare them across groups[5–8]. Since several pharmaceutical companies are working on novel treatments against influenza A including immunotherapeutic approaches[9], our analysis can inform future trials of potential new influenza treatments. The models are most useful for the planning of phase I studies, but more general conclusions can also be drawn for phase II and III studies.

## Materials and Methods

**Fig 1:**
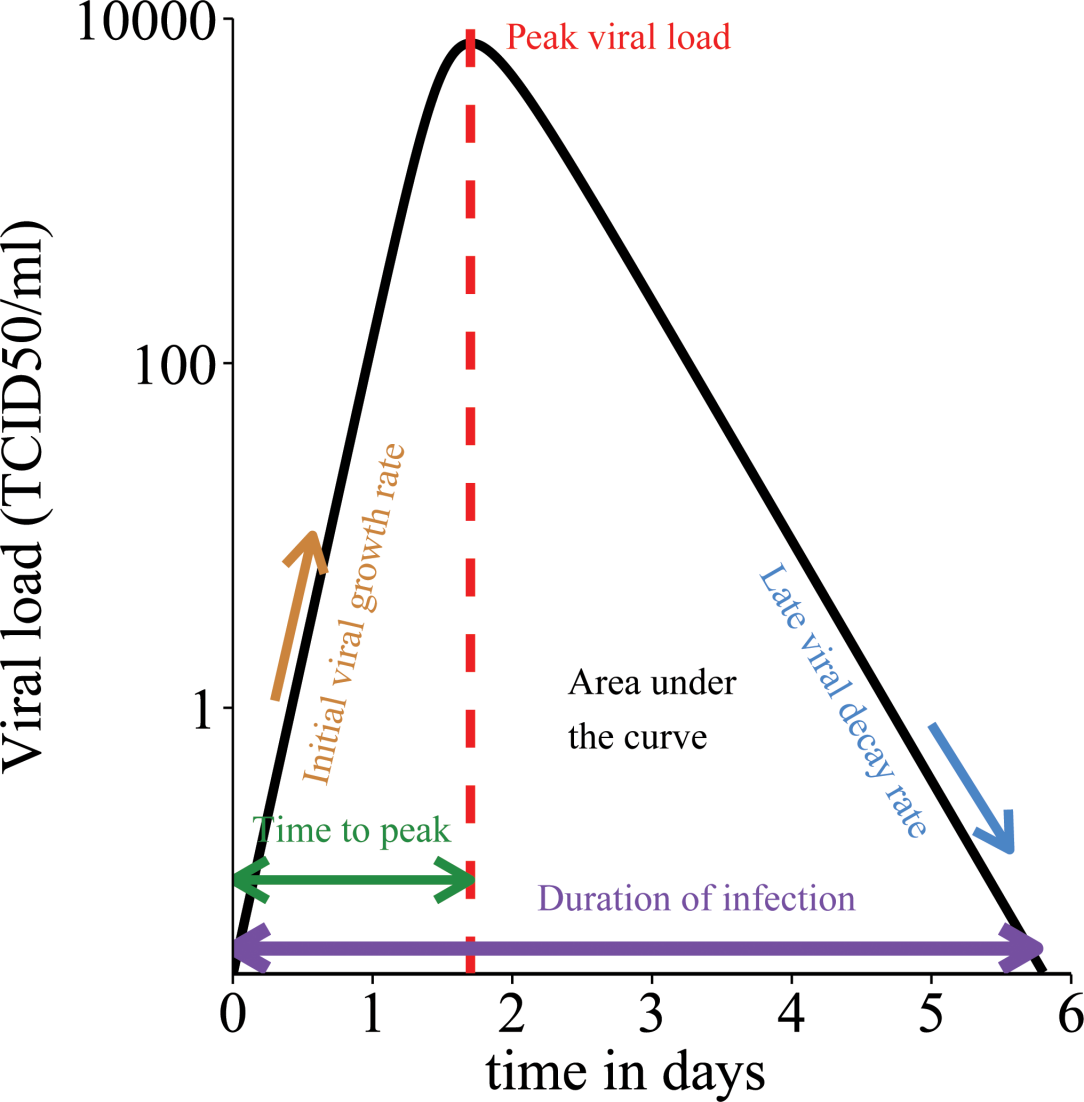
Schematic illustration of key infection-related quantities that define the course of acute viral infections. Black line: viral load curve over time. Filled area indicates viral load area under the curve (AUC). X-axis: time in days post infection. Y-axis: log_10_ viral load (TCID_50_/ml).

In prior research we have analysed the properties of a simple differential equation model of viral load dynamics in influenza A infection and a number of measurable infection-related quantities which can be derived from this model (Fig 1)[10]. The model has been developed from a standard model [11], by assuming that the viral dynamics are much faster than those of infected cells. As we do not have quantitative information on the dynamics of infected cells, this is a sensible assumption that allows us to reduce the number of unknown parameters in the system. The model is general enough to be applicable to other acute viral infections. The analytical properties of this model and the derivation of the infection-related quantities have been discussed elsewhere [10]. In brief, the dynamics of the model are given by the following equations:

$$\frac{dT}{dt}=-\beta TV$$

$$\frac{dV}{dt}=r\beta TV-\gamma V$$

where *T* is the number of epithelial target cells susceptible to viral infection, *V* is the viral load (measured in TCID_50_/ml), β is the infection rate of target cells, *r* is the virus production rate, and γ is the virus death or clearance rate which encompasses the action of specific and non-specific immune mechanisms. The infection-related quantities that can be derived from this model and their interpretations are listed in Table 1. The analytical expressions of the quantities are given in S1 Table (details in [10]).

**Table 1:** Infection-related quantities derived from a simple viral dynamics model that can improve the assessment of therapy against acute viral diseases[10].

Infection-related quantity	Interpretation	Practical use
Basic reproductive ratio (R_0_)	Mean number of cells infected by one primary infected cell. If R_0_>1 the infection will establish in the patient.	Determine if treatment can stop infection (bring R_0_ below 1)
Area under the viral load curve (AUC)	Total amount of virus shed over the time of infection; indicates infectiousness and severity of infection.	Determine if treatment reduces infectiousness and severity of infection
Initial viral growth rate	Rate at which virus multiplies in the early stage of the infection	Determine if (prophylactic) treatment reduces viral growth
Late viral decay rate	Rate at which virus is cleared towards the end of the infection	Determine if treatment increases rate of late viral decay
Peak viral load	Maximum viral load; indicates severity of infection	Determine by how much treatment reduces viral load peak
Time to peak viral load	Time required to reach peak viral load	N/A
Duration of infection	Time from onset of infection to full clearance; in practice measured as time from onset to viral load fall under detection limit	Distinguishes short-lived from chronic infections; determine by how much treatment shortens duration of infection
Generation time	Speed at which infection spreads in population	Determine if treatment slows down speed at which infection spreads
Fraction of dead cells at end of infection	Indicates tissue damage caused by infection; measure of severity of infection	Determine if treatment reduces tissue damage

We fitted the model to viral load data of nine patients from the placebo groups of volunteer challenge studies conducted by Roche as part of the original Oseltamivir trials, using Markov Chain Monte Carlo (MCMC) methods [12]. All volunteers were screened for HI titre to exclude pre-existing immunity against the challenge strain. We estimated the parameter values for each patient. See S1 File for a more detailed description of the data and fitting procedure. We used the parameter estimates to determine the infection-related quantities for each patient. We assessed the variability of the quantities among patients by calculating the coefficients of variation (CV).

In addition to viral load measurements, the data also included temperature measurements and total symptom scores (Jackson score [13]) for each patient at different time points. We calculated pairwise correlations (Pearson’s r) between individual viral load and temperature measurements over time, and between individual viral load and symptom score measurements over time. We also calculated pairwise correlations between each of the nine infection-related quantities, and between the infection-related quantities and the area under the curve (AUC) of the total symptom scores and the temperature area under the curves. The latter two quantities measure the total symptom severity and temperature increase over the entire course of infection (the total symptom score AUC is the AUC over all Jackson scores at each measured time point). We determined the statistical significance of the correlations with two different correction methods for multiple comparison (11 comparisons in total), the Bonferroni method and the Benjamini-Hochberg method [14].

Written informed consent was obtained from each participant in a form approved by the institutional review boards of the University of Virginia, Charlottesville, and the University of Rochester, Rochester, NY, and subjects were compensated for participation. The review board of the named institution has approved the study in which the data we used was collected.

## Results

**Fig 2:**
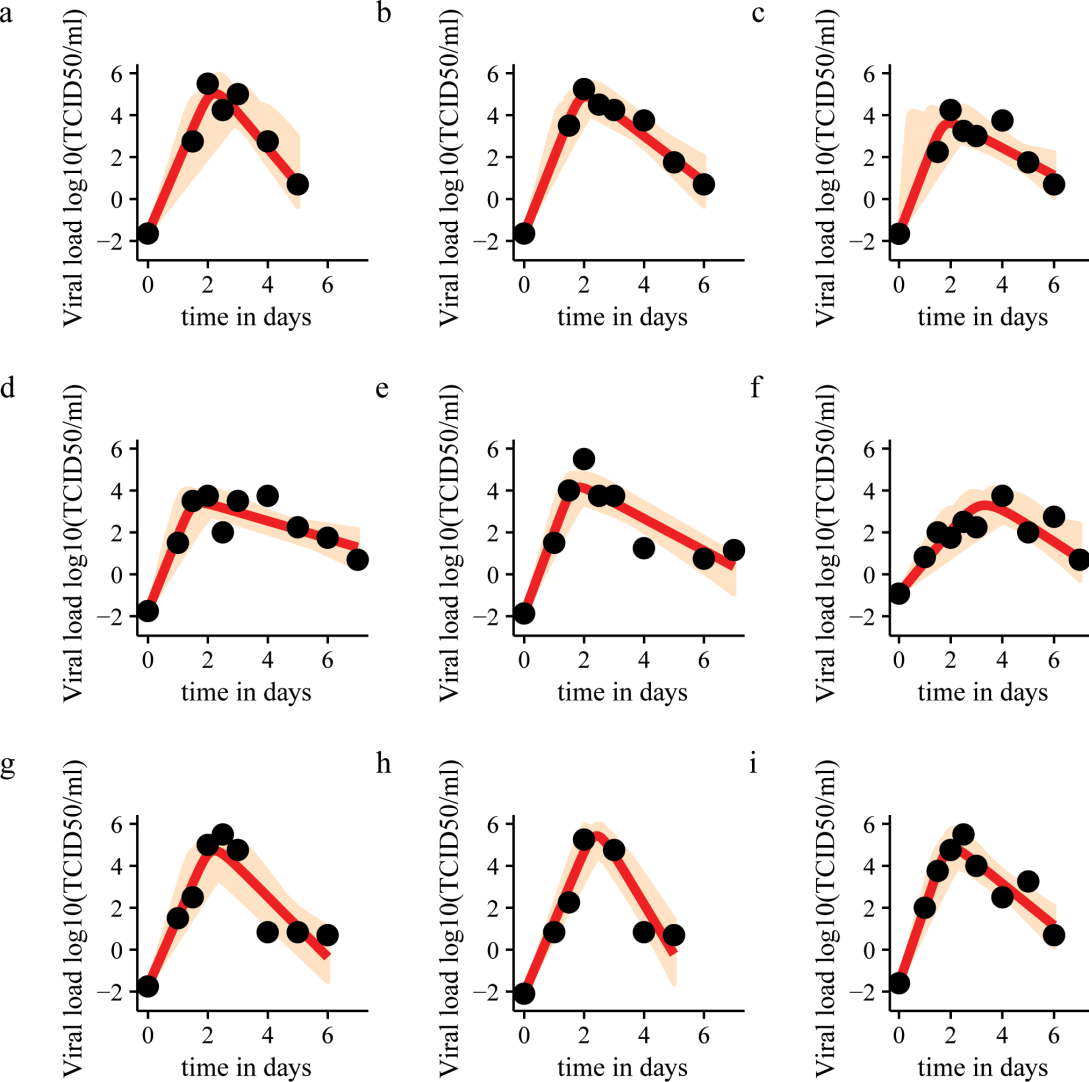
Model fit to data for nine patients. Black dots: viral load data points. Red line: model fit based on median estimates. Light brown area: sample from posterior distributions of viral dynamics. Patients 2 and 3 have a missing data point at day 1. Accordingly, there is great uncertainty about the time of the viral load peak, as illustrated by the sample from the posterior distribution.

Fig 2 shows the model fits for nine patients. The parameter estimates are shown in S2 Table. Compared to the 95% credible intervals of individual parameter estimates, the variation among patients in the infection-related quantities shown in Table 2 is relatively small. This is not surprising considering that the volunteers in the original challenge study were all young, healthy adults from a narrow age band (18–27 years). The greatest variation among individuals occurs in peak viral load (CV = 1.26) and viral load area under the curve (AUC) (CV = 1.19) (see S3 Table for all coefficients of variation.) Temperature significantly correlates with viral load (r = 0.328, p = 0.0025). Total symptom scores also correlate with viral load (r = 0.233, p = 0.03385). When we determined the correlations between infection-related quantities using the Bonferroni correction, the following correlations were significant: *R*_*0*_ and the duration of infection (r = 0.947, p_bonferroni_ = 5.902x10^-3^), viral load AUC and peak viral load (r = 0.9994, p_bonferroni_ = 7.068x10^-10^), viral load AUC and late viral decay rate (r = 0.9509, p_bonferroni_ = 4.514x10^-3^), time to peak viral load and generation time (r = 0.9644, p_bonferroni_ = 1.487x10^-3^), time to peak viral load and initial viral growth rate (r = -0.9459, p_bonferroni_ = 3.384x10^-3^), and peak viral load and late viral decay rate (r = 0.9601, p_bonferroni_ = 2.212x10^-3^). It should be noted that the infection-related quantities are related to each other via the model parameters, which explains the very high correlations.

**Table 2:** Values of infection-related quantities of nine patients based on median estimates of parameters and 95% credible intervals.

Patient number	#1	#2	#3	#4	#5	#6	#7	#8	#9
**Basic reproductive ratio (R**_**0**_**)**	2.33[1.279, 4.026]	4.00[2.497, 15.29]	5.52[2.451, 43.76]	9.60[5.309, 21.85]	6.00[4.170, 8.987]	2.10[1.079, 6.483]	2.78[1.840, 4.092]	1.63[1.240, 2.428]	4.36[2.717, 7.321]
**Area under the viral load curve (AUC)**	79665[19394, 367729]	67364[18814, 262194]	5892[1838, 22013]	4111[1652, 11934]	14966[4760, 50487]	3448[1173, 27139]	42101[11496, 157812]	208096[47773, 940812]	65845[18951, 241242]
**Initial viral growth rate**	7.39[5.588, 9.406]	8.17[6.158, 23.36]	7.06[4.674, 31.17]	8.24[5.627, 11.87]	8.87[6.764, 11.40]	3.12[2.116, 3.417]	7.38[5.756, 9.193]	7.76[6.419, 9.267]	8.18[6.178, 10.63]
**Late viral decay rate**	5.59[2.745, 21.68]	2.73[1.549, 4.561]	1.53[0.630, 3.614]	0.97[0.486, 1.569]	1.78[1.216, 2.444]	2.15[2.124, 4.976]	4.18[2.649, 7.410]	12.34[5.915, 29.56]	2.44[2.432, 2.453]
**Peak viral load**	94472[20448, 438369]	74352[17677, 339935]	4592[1112, 22092]	2535[816, 9361]	14175[3817, 54851]	1507[489, 7210]	49530[11883, 206486]	250118[54944, 1154068]	70852[69555, 71654]
**Time to peak viral load (days)**	2.18[1.907, 2.488]	2.02[0.778, 2.337]	2.03[0.449, 2.531]	1.73[1.305, 2.140]	1.77[1.500, 2.023]	3.28[2.541, 3.774]	2.15[1.918, 2.384]	2.29[2.112, 2.449]	2.00[1.984, 2.010]
**Duration of infection**	4.69[3.561, 5.655]	5.84[5.126, 7.339]	6.39[4.799, 10.93]	8.15[6.321, 13.03]	6.29[5.469, 7.685]	5.56[3.807, 9.863]	4.95[4.443, 5.571]	4.04[3.288, 4.665]	6.16[5.344, 7.773]
**Generation time (days)**	2.55[2.008, 3.676]	2.09[0.784, 2.641]	2.07[0.463, 4.016]	1.73[1.327, 2.168]	1.78[1.502, 2.066]	4.02[2.573, 6.050]	2.40[2.004, 2.994]	3.09[2.488, 3.669]	2.05[1.688, 2.555]
**Fraction of dead cells at end of infection**	0.919[0.7779, 0.9833]	0.983[0.9305, 1.0000]	0.996[0.9275, 1.0000]	1.000[0.9952, 1.0000]	0.998[0.9855, 0.9999]	0.9000.7289, 0.9985]	0.946[0.8718, 0.9843]	0.843[0.7692, 0.9259]	0.988[0.9433, 0.9993]

When we used the Benjamini-Hochberg correction, three additional correlations were significant: R_0_ and total symptom score AUC (r = 0.8613, p_BH_ = 7.374x10^-4^), fraction of dead cells at the end of infection and duration of infection (r = 0.8025, p_BH_ = 0.01961), generation time and initial viral growth rate (r = -0.8693, p_BH_ = 0.01834). Here R_0_ and total symptom score AUC are independent, whereas the fraction of dead cells and the duration of infection, as well as the generation time and initial viral growth rate are dependent on each other via the viral dynamics model. See Table 3 for all correlations.

**Table 3:** Pairwise correlations between infection-related quantities, between infection-related quantities and temperature area under the curve, and between infection-related quantities and symptom score area under the curve. Correlations that are significant using the Bonferroni correction for multiple comparisons are indicated by an asterisk. Correlations that are significant using the less stringent Benjamini-Hochberg correction are indicated by a diamond. Values below the diagonal show Pearson’s correlation coefficient, values above the diagonal show uncorrected p-values for each correlation. AUC_S_: total symptom score area under the curve. AUC_T_: temperature area under the curve. R_0_: basic reproductive number. AUC_V_: viral load area under the curve. FDC: fraction of dead cells at end of infection. t_peak_: time to peak viral load. V_peak_: peak viral load. t_g_: generation time. IGR: initial viral growth rate. LDR: late viral decay rate. D: duration of infection.

	AUC_S_	AUC_T_	R_0_	AUC_V_	FDC	t_peak_	V_peak_	t_g_	IGR	LDR	D
**AUC**_**S**_		0.103	2.85E-3 ◊	0.151	0.183	0.216	0.163	0.186	0.497	0.227	0.0170
**AUC**_**T**_	0.5782		0.143	0.0747	0.251	0.957	0.0739	0.734	0.840	0.0993	0.0903
**R**_**0**_	0.8613 ◊	0.5287		0.121	0.0145	0.0627	0.119	0.0282	0.238	0.0725	1.07E-4 ◊ *
**AUC**_**V**_	-0.5203	-0.6204	-0.5544		0.0312	0.973	1.28E-11 ◊ *	0.524	0.536	8.21E-5 ◊ *	0.0333
**FDC**	0.4876	0.4275	0.7735	-0.7126		0.0678	0.0266	7.88E-3	0.232	0.0116	9.24E-3 ◊
**t**_**peak**_	-0.4576	-0.0214	-0.6413	0.0135	-0.6321		0.958	2.70E-5 ◊ *	6.15E-5 ◊ *	0.696	0.254
**V**_**peak**_	-0.5081	-0.6216	-0.5572	0.9994◊ *	-0.7266	0.0209		0.506	0.554	4.02E-5 ◊ *	0.0309
**t**_**g**_	-0.4844	-0.1324	-0.7215	0.2457	-0.8118	0.9644 ◊ *	0.2563		2.33E-3 ◊	0.289	0.109
**IGR**	0.2616	-0.0790	0.4382	0.2389	0.4436	-0.9549 ◊ *	0.2289	-0.8693◊		0.845	0.604
**LDR**	-0.4474	-0.5832	-0.6240	0.9509◊ *	-0.7886	0.1524	0.9601 ◊ *	0.3981	0.0766		0.0116
**D**	0.7620	0.5960	0.9470◊ *	-0.7067	0.8025 ◊	-0.4256	-0.7135	-0.5708	0.2011	-0.7886	

**Fig 3:**
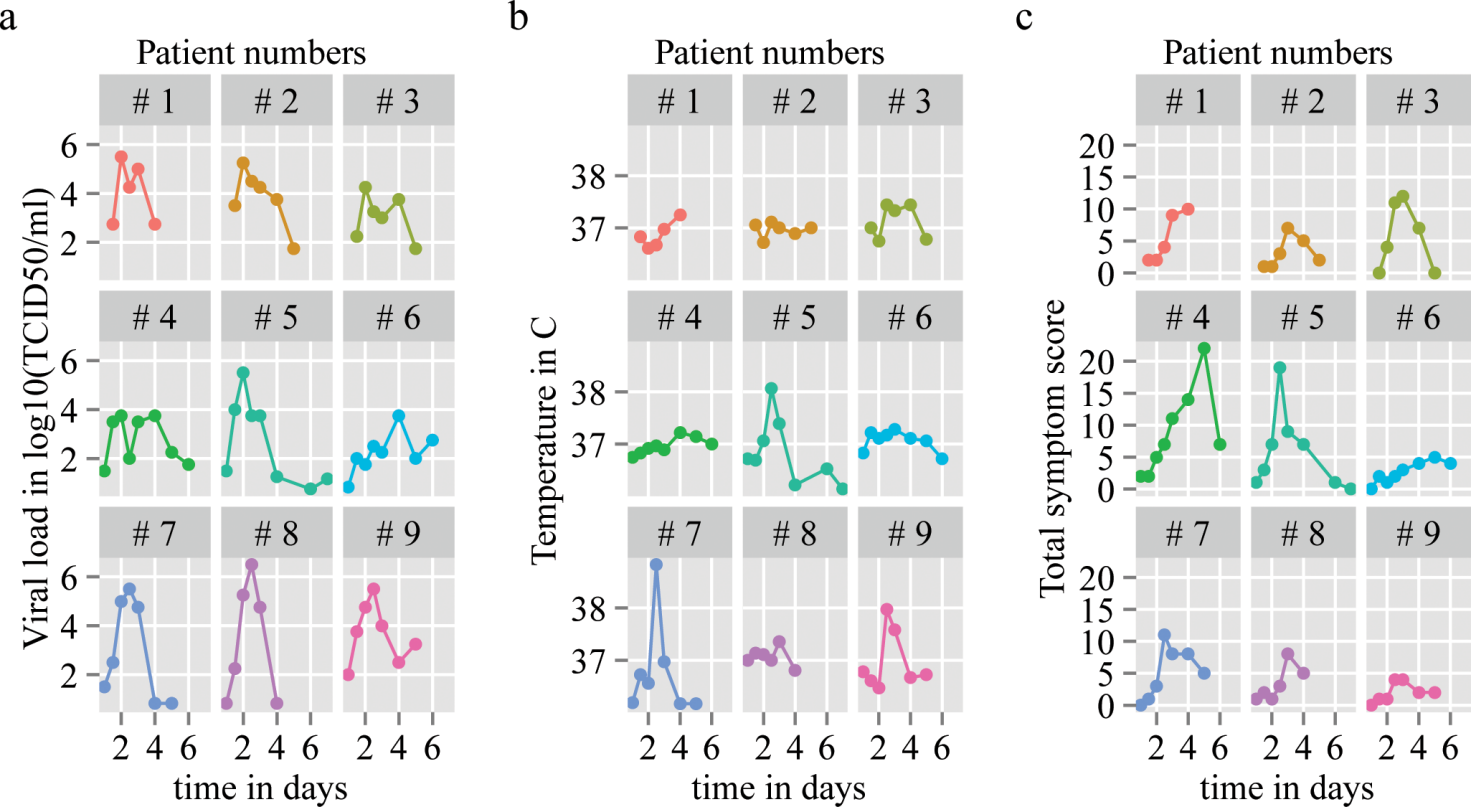
Viral load curves, temperature curves and total symptom score curves of nine patients.

Even though the association is not statistically significant, we observe that Patients 3, 4 and 5 have the highest fraction of dead cells and also the highest symptom scores AUC (Table 2, Fig 3).

## Discussion

Our parameter estimates are of the same size as previous estimates from studies using comparable models (for example [11]). As the parameters in these models tend to be highly correlated, the uncertainty around individual parameter estimates is generally large. Our analysis is limited by the small sample size which, however, is not unusual in studies fitting viral dynamics models.

Our estimates of the basic reproductive number (*R*_*0*_) tend to be lower than previously reported results [11,15]. Some of these published estimates were derived from tissue culture experiments, where, in the absence of any immune response, R_0_ is likely to be higher. The viral strains used also differed in the various studies (influenza A Texas/36/91 (H1N1) in our study, Hong Kong/123/77 (H1N1) in [11], and Albany/1/98 (H3N2) in [15]). As some viral strains may disseminate faster through infected tissue than others, infection by such strains may be associated with higher *R*_*0*_ values. Moreover, some of the earlier studies consider a latent infection stage which can increase estimates of *R*_*0*_. We did not consider a latent stage, as the data did not contain enough information to justify additional parameters.

In our analysis, peak viral load and viral load AUC are the most variable infection-related quantities. They were also strongly correlated, indicating that viral load AUC is mainly determined by peak viral load. High variability in AUC is thought to reflect high variability in infectiousness among individuals.

We found that viral load correlates significantly with body temperature and total symptom scores, but the correlations were not very strong. This may indicate that, although viral load is ultimately responsible for infection-related illness, other processes, for example the immune response, cause symptoms. Alternatively, the reporting of symptoms may be rather subjective and therefore difficult to quantify accurately.

We found that *R*_*0*_ has a significant positive correlation with the duration of infection and the total symptom score AUC. Consequently, *R*_*0*_ may be interpreted as an indicator for disease severity.

Viral load AUC positively correlates with peak viral load and with the late viral decay rate. Peak viral load correlates positively with the late viral decay rate. This suggests that infections with a high peak viral load tend to decline rapidly after the peak (intense, but short infection), whereas infections with a low peak viral load tend to decline more slowly (mild, but extended infection).

The fraction of dead cells at the end of infection correlates positively with the infection duration. This means that, according to our model, longer infections cause more severe tissue damage. In addition, we observed that the patients with the highest fraction of dead cells at the end of infection have the greatest total symptom score AUCs. A possible interpretation may be that symptoms are at least partly caused by tissue damage. The inter-individual variability in the fraction of dead cells, however, is very low (CV = 0.05788, see S3 Table).

Both time to peak viral load and generation time were negatively correlated with initial viral growth. This means that an infection caused by slowly reproducing virus spreads more slowly and has a later peak than an infection caused by faster reproducing virus.

With a bigger sample size, more correlations between infection-related quantities may be found to be significant. For example, in coronavirus infection, a shorter incubation time is associated with more severe disease [16].

**Fig 4:**
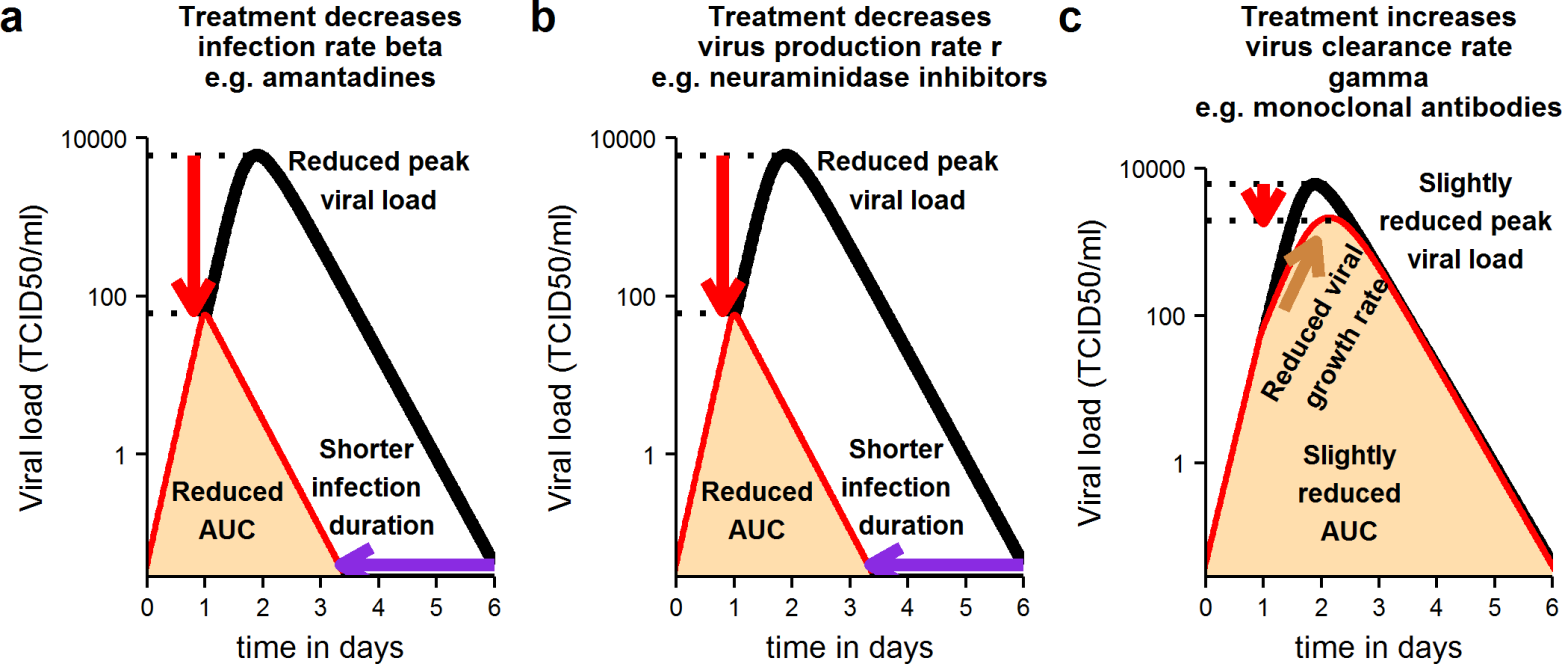
Schematic illustration of changes expected in key infection-related quantities in treated versus untreated individuals (treated before time of peak viral load on day 1). Treatments acting on the infection rate or viral production rate have a strong impact on infection, if given before the time of peak viral load (a, b). They reduce peak viral load, duration of infection and viral load area under the curve (AUC). Treatments acting on virus clearance have a weaker effect on the course of infection, if given before the time of peak viral load (c). Black line: viral load curve in untreated infection. Red line: viral load curve in treated infection. Fill indicates viral load area under the curve in treated infection.

**Fig 5:**
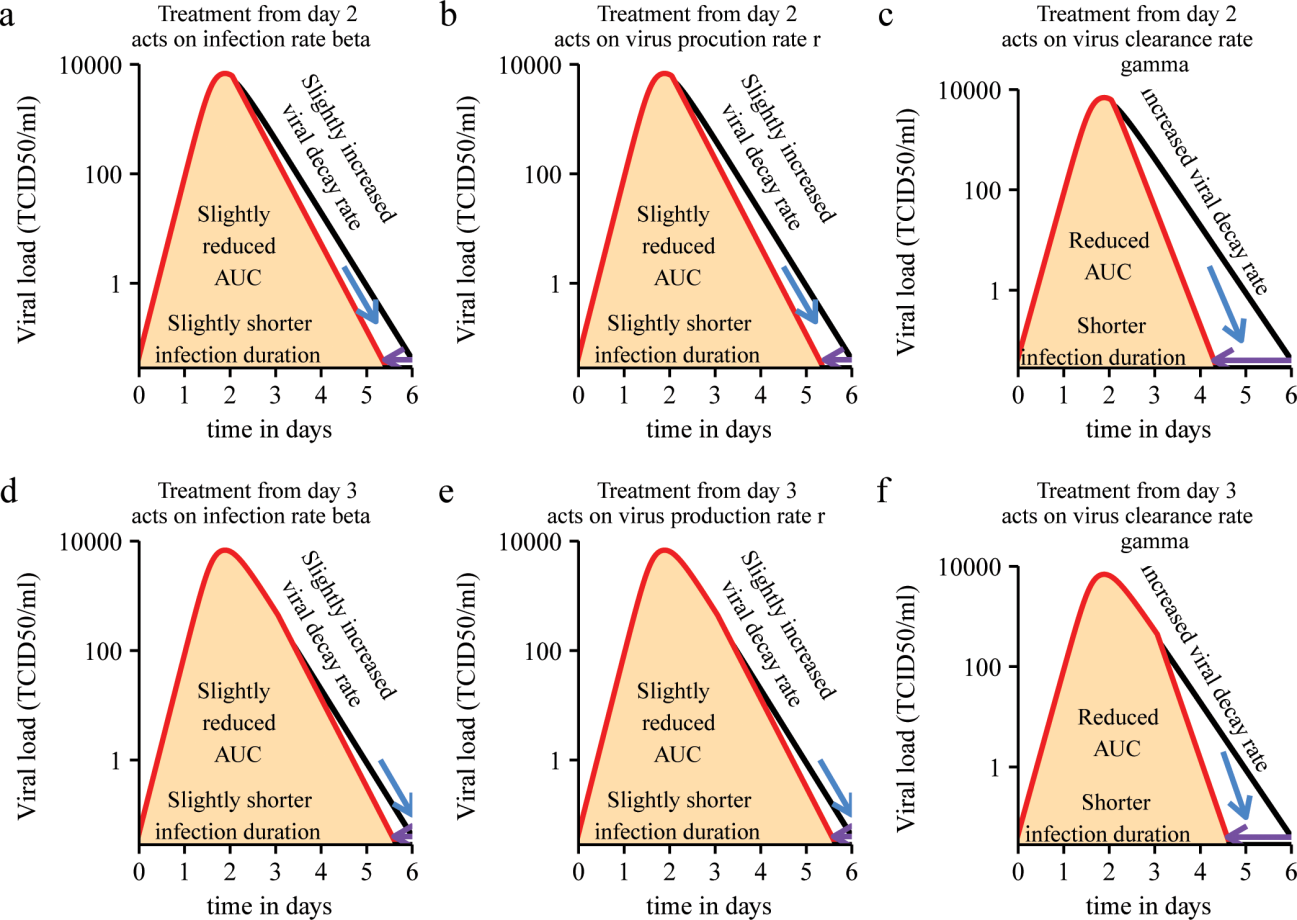
Schematic illustration of changes expected in key infection-related quantities in treated versus untreated individuals (treated after time of peak viral load–days 2, 3). After the peak treatments acting on the infection rate or virus production rate have a small to negligible effect on infection-related quantities (a, b, d, e). After the peak treatments acting on the virus clearance rate have a bigger impact on infection-related quantities (c, f). Black line: viral load curve in untreated infection. Red line: viral load curve in treated infection. Fill indicates viral load area under the curve in treated infection.

Clinical researchers conducting trials of candidate therapies for acute viral infections should be most interested in how infection-related measures change upon treatment. In Figs 4 and 5 we show schematically what changes to expect in different infection-related quantities following treatment, (for a thorough sensitivity analysis see [10]).

We consider treatments that act on the infection rate (in the case of influenza A, for example, amantadines [17]), the viral production rate (neuraminidase inhibitors against influenza A [18]), and the viral clearance rate (monoclonal antibodies against influenza A are thought to act in this way [19]). We consider treatments that are given on days 1, 2 and 3 post infection.

As we derived in [10], treatments that act on viral growth parameters (infection rate and viral production rate) are most effective, when they are given before the time of peak viral load (day 1). They can greatly reduce peak viral load, shorten the duration of infection and reduce the viral load AUC (Fig 4A and 4B). In contrast, after the peak (days 2 and 3), a small to negligible effect on the duration of infection and the viral load AUC is observed (Fig 5A, 5B, 5D and 5E). Our results agree with those from a similar analysis by Kamal et al. [20].

Since patients with acute viral infections typically present at clinics after the time of peak viral load, our model suggests that it will be difficult to detect the impact of a treatment that acts on the infection rate or viral production rate. This may be one of the reasons why different studies have shown ambiguous results on the efficacy of neuraminidase inhibitors for the treatment of influenza A [5–7].

According to our model, when treatments that act on the virus clearance rate are given before the time of peak viral load, they can slow down viral growth and reduce the viral load peak and AUC (Fig 4C). Shortly after the peak, their impact on viral decay and infection duration could be much stronger than that of treatments acting on viral growth parameters (Fig 5C and 5F).

Our predictions strongly depend on the assumption that the infection is target-cell-limited. In infections that are not target-cell-limited and where immunity plays a greater role in reducing viral load, treatments acting on viral growth parameters are expected to have a much stronger effect after the viral load peak.

The choice of endpoint in a clinical trial depends in part on the therapeutic goal. If the goal is to reduce transmission of an infectious agent, the viral load AUC, a measure of infectiousness, is a sensible endpoint. If the goal is to clear infection in a patient, other endpoints may be more useful. Based on our analyses, we make the following recommendations on clinical protocols for the assessment of treatments for acute viral infections with the goal to clear infection in a patient. Our general conclusions hold for all trial phases, but some of our suggestions concerning practical implementation may only be applicable in phase I studies.

When a treatment is given before the time of peak viral load the viral load AUC is commonly used as a virological endpoint. One of the reasons for this is that the AUC is affected by the peak viral load and the duration of infection, and therefore contains more information about the course of infection than either of these quantities on their own. Consequently, the viral load AUC should be the most sensitive indicator of the efficacy of a treatment.

According to our findings, however, the viral load AUC is the infection-related quantity that varies most among individuals. Due to the high natural variability among untreated patients, it should be more difficult to detect a statistically significant difference between treated and control groups. The same consideration holds for the peak viral load which is also highly variable. Conversely, the duration of infection varies much less among individuals (CV = 0.2059). Therefore, besides the reduction in AUC and peak viral load, the duration of infection should be considered as an endpoint.

When a treatment is given after the peak viral load, the AUC is not a very sensitive marker of impact, because the reduction in the total area will be relatively smaller (see Fig 5). The increase in the late viral decay rate and the shortening of the infection duration should be used as alternative endpoints. The duration of infection should be a more sensitive efficacy measure compared to the increase in the late viral decay rate, because the latter is more variable among individuals (CV = 0.9402).

The measured duration of infection will depend on the number of measurements taken and the sensitivity of the viral load assay. It is also easy to miss the peak viral load, if the measurement intervals are spaced too far apart. This will affect the estimate of the viral load AUC. We recommend taking at least two to three measurements per day, if possible (mainly in phase I studies). This is partly because the reduction in infection duration in treated individuals is typically not more than one day (Fig 5). If only one measurement per day is taken, it will be difficult to resolve the differences in infection duration between treated and untreated groups. The same rationale holds for measuring the viral decay rate.

Taking more frequent viral load measurements is essential for fitting mechanistic models of acute viral infections to data. In order to fit the model we introduced above, one needs a minimum of at least five data points taken at different times [21]. In particular, parameter estimation requires early data points before the time of the peak. Such data can normally only be collected in volunteer challenge studies and possibly prospective cohort studies (for example [22]).

The lower the measurement error and the higher the sensitivity of an assay, the more accurate the data. Determining the distribution of the measurement error of a given assay can improve statistical inference. This can be achieved by running replicate measurements at each step of the assay on the same sample and from different samples taken from the same patient at one time point. In the case of influenza A, the most common assays are RT-qPCR and TCID_50_ [23,24]. Information on the error distribution of the assay can be incorporated in the model fitting procedure to obtain more reliable estimates of infection-related quantities.

We suggest a number of laboratory experiments that could help to improve estimates of model parameters and disease markers. Parameters that can be measured independently can be set to a fixed value or allow the definition of better prior distributions during the model fitting procedure, so that the remaining free parameters can be estimated with greater accuracy.

For example, the decay rate of influenza A virus has been measured previously in micro-carrier culture [25]. Viral culture medium, however, does not contain any components of the immune system. Consequently, the measured viral decay rate is much lower than inside the human host (provided he or she is not immunosuppressed). It should be possible to culture virus and add serum taken at different times post infection from previously infected individuals to the culture medium. This would give independent and more realistic estimates of the virus decay rate in its natural environment.

The virus production rate per cell can be estimated by determining the total amount of virus produced in a culture system and dividing it by the total amount of host cells in the system [25]. The virus production rate may be affected by the innate immune response, as may be the virus clearance rate. It would likely be viral strain- and cell type-specific and would have to be measured for all strains against which a treatment was to be tested and for several respiratory epithelial cell types. The half-life of cells infected by different strains can also be measured using common apoptosis and cytotoxicity markers [26,27].

These experiments may not be cheap, and may be rather complex. In order to supplement and validate data obtained from tissue culture systems, animal models may prove useful. In particular, it may be possible to obtain data on the dynamics of infected cells from serial autopsies from sacrificed animals. The more components of the virus life cycle and the human immune response can be measured independently the more complex and detailed models can be built of viral infection. Hence better assessments of therapy impact can be made. Compared to the cost of failed clinical trials, the cost and endeavour of additional laboratory experiments should be worth the attempt.

## Supporting Information

S1 File Markov Chain Monte Carlo (MCMC) parameter estimation. Description of the parameter estimation procedure.
(DOCX)

S1 Table Analytical expressions of infection-related quantities for acute viral infections. (DOCX)

S2 Table Median estimates of model parameters and 95% credible intervals. (DOCX)

S3 Table Coefficients of variation of infection-related quantities. (DOCX)

S4 Table Model of acute viral infections with terms representing treatment. (DOCX)
