# Supplementary material for: How Can Viral Dynamics Models Inform Endpoint Measures in Clinical Trials of Therapies for Acute Viral Infections?
Source: PLoS One. 2016 Jul 1;11(7):e0158237. doi: 10.1371/journal.pone.0158237 (PMC4930163; doi:10.1371/journal.pone.0158237)
Supplement: S1 File — Description of the parameter estimation procedure. (DOCX) [file pone.0158237.s001.docx]

**S1 File – Markov Chain Monte Carlo (MCMC) parameter estimation**

**Data description**

We estimated the parameters of the model summarised in the main text ^1^, using viral load data (in TCID_50_/ml) of individuals from the placebo group of two volunteer challenge studies (study numbers PV15615, PB15616) conducted by Roche as part of the original oseltamivir trials ^2^. In this study, healthy adult volunteers (aged 18-27 years) were challenged by intranasal inoculation with 10^6^ TCID_50_ human influenza A virus (strain A/Texas/36/91). Viral load data was measured for each patient by nasal lavage on days 1, 1.5, 2, 2.5, 3 and daily up to day 9 thereafter.

In order to estimate the parameters, we need at least five independent data points taken at five different times ^3^. Therefore, we only analysed data from patients with five or more data points. We excluded patients that had no measurements during the first three days, as preliminary analyses had shown that the model cannot be fitted to these data with precision. These requirements left datasets from nine patients (original number 25) in the Roche trial. We estimated the parameters for each of them independently.

There was no information about the limit of detection of viral load in our dataset. Hence we assumed the limit of detection to be the smallest measurement value in the dataset (0.7 TCID50/ml). When at the right end of the viral load curve two or more data points had the value of the assumed limit of detection, we excluded all apart from the first.

In addition to viral load measurements, the data also included temperature measurements and total symptom scores (Jackson scores ^4^) for each patient.

**MCMC**

**Reparametrisation of the model**

In the original model notation the parameters β and *r* are highly correlated. For the estimation of the parameters, we reparametrised the model and substituted the product term β*r* in the equation describing the viral load dynamics by *r’*:

$$\frac{dT}{dt}= -\beta TV$$

$$\frac{dV}{dt}=r'TV- \gamma V$$

We estimated the values of β, *r’* and γ, and calculated the median and confidence intervals for *r* from the traces of β and *r’*.

**Prior distributions**

Independent prior distributions were chosen (Table A) for each parameter (except the initial number of target cells T0 which was fixed at 4 x 10^8^, the estimated number of cells in the human upper respiratory tract ^5^).

Table A: Prior distributions for MCMC parameter estimation

| Parameters | Explanation | Prior Distributions | Units |
| --- | --- | --- | --- |
| *V_0_* | Initial viral load | Exponential (mean=100) | TCID_50_ mL^-1^ |
| β | Infection rate of target cells | Uniform (0-0.003)* | TCID_50_ mL day^-1^ |
| *r’* | Virus production rate | Uniform (0-0 .0000001) | day^-1^ |
| γ | Virus clearance rate | Exponential (mean=100) | day^-1^ |
| *T_0_* | Initial number of target cells | Fixed to 4 x 10^8^ | cell |

* except for patients 3 and 4: (0-0.01). Additional experimental measurements may help define better prior distributions, as explained in the Discussion.

As no independent information on the parameter values was available, we chose flat exponential priors for *V_0_* and γ. However, we could not use unrestricted priors for β and *r*, because, in this case, both values tended to be unrealistically high. Therefore, we chose uniform distributions from 0-0.03 for β and 0-0.0000001 for *r’*. For patients 3 and 4, we chose the prior for β to be uniformly distributed over 0-0.01. The uniform priors were chosen so that the traces still converged, but would not escape to unrealistically high values.

**Likelihood**

The viral load data had been measured by the median tissue culture infective dose (TCID_50_) assay. Hence we assumed that the measured viral load were lognormal distributed around the true viral loads ^6^. Preliminary analyses had shown that the assumed standard deviation of the lognormal distribution does not affect the parameter estimates. The standard deviation in our analyses was set to 0.3 log_10_ units.

**Sampler and proposal distribution**

The parameters were updated individually using random-walk Metropolis-Hastings sampling. At each sampling step, the standard deviation of the normal proposal distribution was adjusted by an adaptive scale factor depending on the acceptance ratio of the sampler.

For each dataset 8.1 x 10^5^ sampling iterations were performed. The first 10^5^ iterations were discarded as a burn-in phase. During sampling only every 100th iteration was recorded to avoid autocorrelation.

**Implementation**

The MCMC algorithm was implemented with the package PyMC 2.3.4 for Python 2.7 ^7^. The ordinary differential equation model was implemented using the scipy.integrate function ode (method ‘lsoda’, maximum number of iterations per integration time step 1000).

**Validation**

Convergence of the estimation procedure was assessed by visually inspecting the parameter traces. The posterior distributions were markedly different from the prior distributions. While the prior distributions were chosen to be flat and uninformative, the posterior distributions were bell-shaped. For example see Figure A.


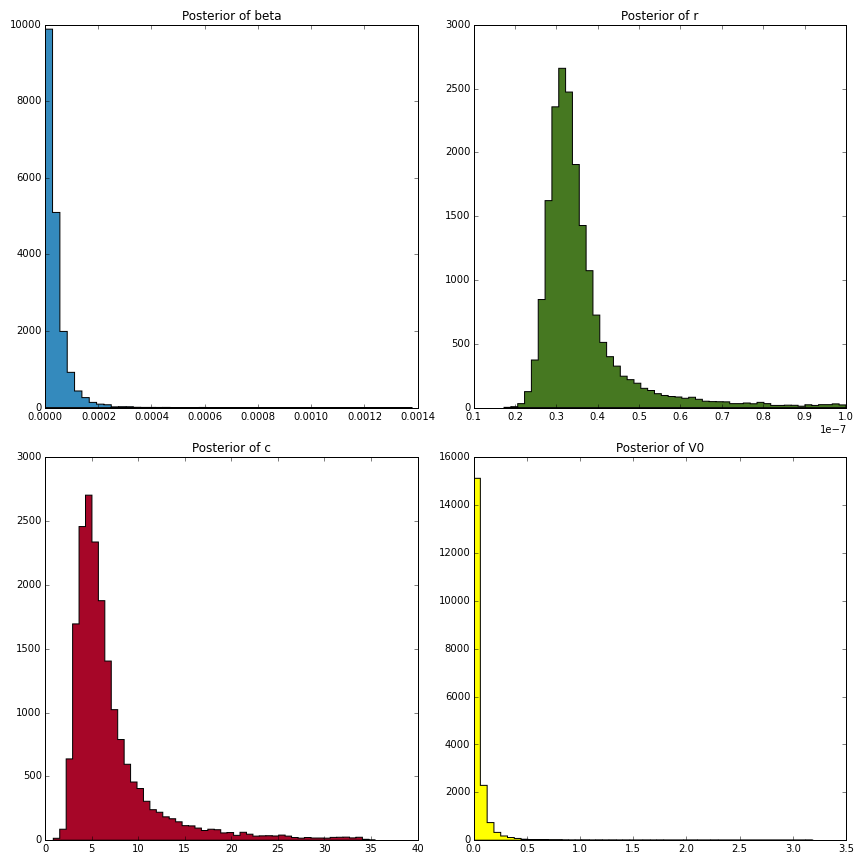


**Figure A: Example of posterior distributions of estimated parameters (Patient 1).**

1. Hadjichrysanthou C, Cauët E, Lawrence E, Vegvari C, De Wolf F, Anderson R. The Use of Mathematical Models to Understand the Within-Host Dynamics of Influenza A Virus Infection and the Impact of Candidate Therapies: From Theory to Clinical Application. *PLoS computational biology* submitted.

2. Hayden FG, Treanor JJ, Fritz RS, et al. Use of the oral neuraminidase inhibitor oseltamivir in experimental human influenza: randomized controlled trials for prevention and treatment. *JAMA : the journal of the American Medical Association* 1999; **282**(13): 1240-6.

3. Miao H, Xia X, Perelson AS, Wu H. On identifiability of nonlinear ODE models and application in viral dynamics. *SIAM review Society for Industrial and Applied Mathematics* 2011; **53**(1): 3-39.

4. Jackson GG DH, Spiesman IG, Boand AV. Transmission of the common cold to volunteers under controlled conditions. I. The common cold as a clinical entity. *AMA Archives of Internal Medicine* 1958; **101**(2): 267-78.

5. Baccam P, Beauchemin C, Macken CA, Hayden FG, Perelson AS. Kinetics of influenza A virus infection in humans. *Journal of virology* 2006; **80**(15): 7590-9.

6. Wulff NH, Tzatzaris M, Young PJ. Monte Carlo simulation of the Spearman-Kaerber TCID50. *Journal of clinical bioinformatics* 2012; **2**(1): 5.

7. Patil A, Huard D, Fonnesbeck CJ. PyMC: Bayesian Stochastic Modelling in Python. *Journal of Statistical Software* 2010; **35**(4): 1-81.
