# Supplementary material for: How Can Viral Dynamics Models Inform Endpoint Measures in Clinical Trials of Therapies for Acute Viral Infections?
Source: PLoS One. 2016 Jul 1;11(7):e0158237. doi: 10.1371/journal.pone.0158237 (PMC4930163; doi:10.1371/journal.pone.0158237)
Supplement: S1 Table — (DOCX) [file pone.0158237.s002.docx]

**S1 Table : Analytical expressions of infection-related quantities for acute viral infections.**

The derivation of these quantities can be found in Hadjichrysanthou et al^1^.

| Infection-related quantity | Formula |
| --- | --- |
| Basic reproductive number (R_0_) | $R_{0 =} \frac{\beta r}{\gamma}T_{0}$ |
| Area under the viral load curve (AUC) | $A= \frac{1}{\beta}\left( w\left( z_{1} \right)+ \frac{\beta}{\gamma}V_{0}+ R_{0} \right)$ , $z_{1}= -R_{0}e^{-R_{0}-\frac{\beta}{\gamma}V_{0}}$  w: Lambert W function $w\left( z_{1} \right)e^{w\left( z_{1} \right)}=z_{1}$ |
| Initial viral growth rate | $g_{init}= r\beta T_{0}- \gamma$ |
| Late viral decay rate | γ |
| Peak viral load | $V_{Peak}= V_{0}+rT_{0}- \frac{\gamma}{\beta}ln(R_{0}e)$ |
| Time to peak viral load | $t_{Peak}= \frac{1}{\gamma- \beta(V_{0}+rT_{0})} ln\frac{\gamma V_{0}}{r\beta T_{0}V_{Peak}}$ |
| Duration of infection | $t_{d}= \frac{1}{\gamma-r\beta T_{t_{2}}}\left( ln\frac{\gamma}{r\beta T_{0}}+r\beta t_{Peak}\left( \frac{V_{0}}{r}+T_{0}- T_{t_{2}} \right)-ln\frac{V_{t_{2}}}{V_{0}} \right)$  Number of uninfected target cells at time t_2_ (when viral load *V* falls below detection limit): $T_{t_{2}}= -\frac{\gamma}{r\beta}w(z_{2})$  $z_{2}= -\frac{r\beta}{\gamma}T_{t_{1}}e^{-\frac{r\beta}{\gamma}T_{t_{1}}+ \frac{\beta}{\gamma}V_{t_{2}}- \frac{\beta}{\gamma} V_{t_{1}}}$  w: Lambert W function $w\left( z_{2} \right)e^{w\left( z_{2} \right)}=z_{2}$  $V_{t_{2}}$was set to the detection limit of the viral load assay (0.7 TICD_50_/ml).  For the approximation of $t_{d}$ we set $T_{t_{1}}$to $T_{0}$ and $V_{t_{1}}$to $V_{0}$ |
| Generation time | $T_{g}= \frac{t_{Peak}}{\beta A} ln\frac{T_{0}}{T_{0}+ \frac{V_{0}}{r}- \frac{\gamma}{r}A}$ |
| Fraction of dead cells at end of infection | $D=1+ \frac{1}{R_{0}}w(z)$, $z= -R_{0}e^{-R_{0}-\frac{\beta}{\gamma}V_{0}}$  w: Lambert W function $w\left( z \right)e^{w\left( z \right)}=z$ |

The expressions for the basic reproductive ratio *R_0_*, the late viral decay rate γ, and the peak viral load are precise. All other expressions are approximations.

**Parameter list:**

*T_0_*: initial number of not infected target cells, *V_0_*: concentration of free virus, β: infection rate of target cells by virus, *r*: virus production rate, γ: virus clearance rate

1. Hadjichrysanthou C, Cauët E, Lawrence E, Vegvari C, De Wolf F, Anderson R. 2016 The Use of Mathematical Models to Understand the Within-Host Dynamics of Influenza A Virus Infection and the Impact of Candidate Therapies: From Theory to Clinical Application. *J. R. Soc. Interface* accepted.
