# Supplementary material for: How Can Viral Dynamics Models Inform Endpoint Measures in Clinical Trials of Therapies for Acute Viral Infections?
Source: PLoS One. 2016 Jul 1;11(7):e0158237. doi: 10.1371/journal.pone.0158237 (PMC4930163; doi:10.1371/journal.pone.0158237)
Supplement: S2 Table — (DOCX) [file pone.0158237.s003.docx]

**S2 Table: Median estimates of model parameters and 95% credible intervals for 9 patients who were infected on day zero but not treated (placebo group).** Parameters have been estimated from placebo group data of Roche trial PV-15615-16 using Markov Chain Monte Carlo Methods. For details of the parameter estimation procedure please refer to S2 File.

|  | **Infection rate β [(TCID_50_/ml)^-1^ x d^-1^]** | **Virus production rate *r* [TCID_50_/(ml x d)]** | **Virus clearance rate* γ [d^-1^]** | **Initial viral load *V_0_* [TCID_50_/ml]** |
| --- | --- | --- | --- | --- |
| **Patient 1** | 2.674 x 10^-5^  [3.556 x 10^-6^, 1.579 x 10^-4^] | 1.246 x 10^-3^  [1.679 x 10^-4^, 1.740 x 10^-2^] | 5.588  [2.745, 21.68] | 2.255 x 10^-2^  [1.888 x 10^-3^, 2.678 x 10^-1^] |
| **Patient 2** | 5.974 x 10^-5^  [1.305 x 10^-5^, 3.009 x 10^-4^] | 4.737 x 10^-4^  [9.230 x 10^-5^, 2.371 x 10^-3^] | 2.731  [1.549, 4.561] | 2.275 x 10^-2^  [1.892 x 10^-3^, 2.616 x 10^-1^] |
| **Patient 3** | 1.161 x 10^-3^  [1.681 x 10^-4^, 8.146 x 10^-3^] | 2.194 x 10^-5^  [4.484 x 10^-6^, 1.481 x 10^-4^] | 1.531  [0.6297, 3.614] | 2.178 x 10^-2^  [1.780 x 10^-3^, 2.628 x 10^-1^] |
| **Patient 4** | 2.394 x 10^-3^  [5.715 x 10^-4^, 8.310 x 10^-3^] | 9.641 x 10^-6^  [2.791 x 10^-6^, 4.127 x 10^-5^] | 0.9660  [0.4857, 1.569] | 1.783 x 10^-2^  [1.637 x 10^-3^, 2.094 x 10^-1^] |
| **Patient 5** | 3.995 x 10^-4^  [1.008 x 10^-4^, 1.514 x 10^-3^] | 6.682 x 10^-5^  [1.641 x 10^-5^, 2.803 x 10^-4^] | 1.780  [1.216, 2.444] | 1.366 x 10^-2^  [1.352 x 10^-3^, 1.426 x 10^-1^] |
| **Patient 6** | 8.914 x 10^-4^  [7.773 x 10^-5^, 2.846 x 10^-3^] | 1.552 x 10^-5^  [4.040 x 10^-6^, 4.949 x 10^-4^] | 2.153  [0.7617, 14.18] | 1.053 x 10^-1^  [1.133 x 10^-2^, 7.249 x 10^-1^] |
| **Patient 7** | 6.163 x 10^-5^  [1.359 x 10^-5^, 2.758 x 10^-4^] | 4.762 x 10^-4^  [9.247 x 10^-5^, 2.543 x 10^-3^] | 4.184  [2.649, 7.410] | 1.749 x 10^-2^  [1.817 x 10^-3^, 1.638 x 10^-1^] |
| **Patient 8** | 6.621 x 10^-6^  [1.216 x 10^-6^, 3.713 x 10^-5^] | 7.746 x 10^-3^  [9.785 x 10^-4^, 6.346 x 10^-2^] | 12.34  [5.915, 29.56] | 8.088 x 10^-3^  [9.526 x 10^-4^, 6.579 x 10^-2^] |
| **Patient 9** | 6.476 x 10^-5^  [1.389 x 10^-5^, 3.127 x 10^-4^] | 4.100 x 10^-4^  [7.933 x 10^-5^, 2.129 x 10^-3^] | 2.443  [1.468, 3.993] | 2.503 x 10^-2^  [2.283 x 10^-3^, 2.536 x 10^-1^] |

* The virus clearance rate encompasses the natural death rates of infected cells and free virus and the action of the immune system in enhancing the clearance of both.
