# Supplementary material for: How Can Viral Dynamics Models Inform Endpoint Measures in Clinical Trials of Therapies for Acute Viral Infections?
Source: PLoS One. 2016 Jul 1;11(7):e0158237. doi: 10.1371/journal.pone.0158237 (PMC4930163; doi:10.1371/journal.pone.0158237)
Supplement: S3 Table — (DOCX) [file pone.0158237.s004.docx]

**S3 Table: Coefficients of variation to assess the variability among patients for each quantity.**

| **Quantity** | **Coefficient of variation** |
| --- | --- |
| R_0_ | 0.591292 |
| Fraction dead cells | 0.057883 |
| Time to peak | 0.211471 |
| Generation time | 0.302254 |
| AUC | 1.189274 |
| Initial growth rate | 0.228665 |
| Late decay rate | 0.940204 |
| Peak viral load | 1.260737 |
| Infection duration | 0.205875 |
