# Supplementary material for: How Can Viral Dynamics Models Inform Endpoint Measures in Clinical Trials of Therapies for Acute Viral Infections?
Source: PLoS One. 2016 Jul 1;11(7):e0158237. doi: 10.1371/journal.pone.0158237 (PMC4930163; doi:10.1371/journal.pone.0158237)
Supplement: S4 Table — (DOCX) [file pone.0158237.s005.docx]

**S4 Table.1: Model of acute viral infections with terms representing treatment.**

| **Treatment acts on** | **Example in influenza** |  |
| --- | --- | --- |
| Infection rate | Amantadines | $\frac{dT}{dt}= -(1- \varepsilon_{1})\beta TV$  $\frac{dV}{dt}=-(1- \varepsilon_{1})r\beta TV- \gamma V$ |
| Virus production rate | Neuraminidase inhibitors | $\frac{dT}{dt}= -\beta TV$  $\frac{dV}{dt}=(1- \varepsilon_{2})r\beta TV- \gamma V$ |
| Virus clearance rate * | Possibly monoclonal antibodies | $\frac{dT}{dt}= -\beta TV$  $\frac{dV}{dt}=r\beta TV-(\gamma-\ln\left( 1- \varepsilon_{3} \right))V$ |
| On all model parameters | Possibly monoclonal antibodies | $\frac{dT}{dt}= -(1- \varepsilon_{1})\beta TV$  $\frac{dV}{dt}=(1- \varepsilon_{1})(1- \varepsilon_{2})r\beta TV-(\gamma-\ln\left( 1- \varepsilon_{3} \right))V$ |

*T*: number of not infected target cells, *V*: concentration of free virus, β: infection rate of target cells by virus, *r*: virus production rate, γ: virus clearance rate

ε_1_: efficacy with which treatment acts on infection rate β; ε_2_: efficacy with which treatment acts on virus production rate *r*; ε_3_: efficacy with which treatment acts on virus clearance rate

* In our model virus is cleared exponentially. The logarithmic term represents the increase in the instantaneous virus death rate after the addition of treatment.
